# Pubertal developmental, body mass index, and cardiovascular autonomic function in children and adolescents with and without autism spectrum disorder: a four-time point accelerated longitudinal study

**DOI:** 10.1186/s11689-025-09602-y

**Published:** 2025-03-19

**Authors:** Rachael A. Muscatello, Meredith Cola, Simon Vandekar, Blythe A. Corbett

**Affiliations:** 1 Department of Psychiatry and Behavioral Sciences, Vanderbilt University Medical Center, 1500 21st Avenue South, Suite 2200, TN Nashville, 37212 USA; 2 Vanderbilt Kennedy Center, Vanderbilt University Medical Center, TN Nashville, USA; 3 Department of Biostatistics, Vanderbilt University Medical Center, Nashville, TN USA; 4 Department of Psychology, Vanderbilt University, TN Nashville, USA

**Keywords:** Heart rate, Variability, Autonomic, Autism, Cardiovascular, Health, Physical, Pubertal development

## Abstract

**Background:**

The Autonomic Nervous System (ANS) regulates ‘automatic’ functions such as heart rate, and alterations may have significant impacts on health outcomes. Cardiovascular measures of autonomic function such as heart rate variability are of interest as biological markers in autism spectrum disorder (ASD). The interplay between the ANS and physical health establishes a need to examine cardiovascular autonomic functioning in youth with and without ASD over development. The current study aimed to identify change in autonomic function and balance across the parasympathetic and sympathetic branches over time as a function of diagnosis, age, pubertal development, and physical health status.

**Methods:**

The study included 244 ASD (*N* = 140) or neurotypical (NT) (*N* = 104) youth, ages 10 to 13 years at enrollment and followed over four years. Resting state autonomic functioning was measured using respiratory sinus arrhythmia (RSA; parasympathetic) and pre-ejection period (PEP; sympathetic). Autonomic balance and regulation were also examined as outcomes. Linear mixed models tested between- and within-group differences in the primary autonomic outcomes as well as the influence of pubertal development, body weight, and medication use.

**Results:**

Baseline models showed diagnostic differences, with lower parasympathetic regulation, in youth with ASD, but no differences were observed for the other three outcomes. Adding body mass index (BMI) percentile and medication use removed the statistically significant diagnostic effect, while both variables were significantly related to lower RSA and overall autonomic regulation. Parasympathetic function (RSA) was stable over age and pubertal stage, while a notable decrease in sympathetic control (increased PEP) was found for age and pubertal stage. BMI percentile at enrollment significantly predicted autonomic function, while change in BMI over time did not.

**Conclusions:**

Minimal research to date has explored physical health (e.g., BMI) and autonomic outcomes in ASD. The current study observed few group differences yet demonstrates important effects of physical health on ANS function in both ASD and neurotypical youth. Findings further emphasize a need to focus on individual traits such as BMI and medication use to elucidate the extent to which autonomic differences are related to health status, irrespective of diagnostic category, across the lifespan.

**Supplementary Information:**

The online version contains supplementary material available at 10.1186/s11689-025-09602-y.

## Background

The Autonomic Nervous System (ANS) regulates visceral organs and ‘automatic’ functions such as heart rate, respiration, or digestion. The ANS is divided into two branches – the parasympathetic (‘rest and relax’) and sympathetic (‘fight or flight’) nervous systems. The branches serve largely differential functions, with the parasympathetic (PNS) acting to conserve energy while the sympathetic (SNS) mobilizes the body to react and respond to environmental stressors. A prime example of these dynamic functions can be observed at the heart, where both the PNS and SNS project onto the sinoatrial node of the heart. The relative contributions of either branch at any given time will influence the beat-to-beat variability of the heart, thereby demonstrating the utility of measuring heart rate variability (HRV) as a marker of cardiovascular autonomic function.

Despite the differential functions of the PNS and SNS, these systems do not act independently and purely reciprocally of the other. The regulation of the ANS and its separate branches fall in a two-dimensional space of reciprocal, co-activation, and co-inhibition response profiles [9, 10]. Cardiovascular autonomic balance (CAB) can be conceptualized as the relative reciprocal balance between the PNS and SNS cardiac control. Cardiovascular autonomic regulation (CAR) represents overall cardiovascular autonomic control, taking into account not only PNS and SNS differences along a one-dimensional continuum, but also accounting for occasions of co-activation and co-inhibition of the two branches [10].

These differences in autonomic control across the whole autonomic space are critical to understanding how changes in autonomic function may influence health and disease (e.g., [10, 65, 81]). In fact, a 2010 review by Thayer and colleagues (2010) showed evidence that decreased HRV was associated with the primary risk factors for heart disease and stroke as defined by the National Heart, Lung, and Blood Institute (2022) [76]. Researchers have suggested that altered HRV may serve as an early predictor of cardiovascular diseases where the process of autonomic imbalance initiates a cascade that ultimately results in increased morbidity and mortality [101]. Indeed, reduced parasympathetic regulation and/or increased sympathetic activation have been reported in patients with history of myocardial infarction (heart attack) and chronic heart failure (e.g., [36, 49, 55, 103]). Therefore, understanding individual differences in ANS function throughout the lifespan could be important to identifying those at risk of significant negative health outcomes as cardiovascular diseases are the leading cause of death globally [112].

It is important to acknowledge that this ANS and physical health relationship is likely bidirectional, as physical health changes can also influence and contribute to future autonomic differences. A prominent example of one proxy for physical health is seen in the impact of body weight and body mass on the ANS [61, 67, 91, 100]. Body mass index (BMI) is a calculation that estimates body fat based on an individual’s height and weight to ascertain weight status (i.e., normal weight, underweight, overweight, or obese) to assess health risks. In youth, BMI percentile can be calculated to represent how a child’s measurements compare to others of the same sex and age. BMI has been correlated with reduced parasympathetic regulation as well as elevated sympathetic activation in adults (e.g., [20, 50, 67, 102]) and in children (e.g., [30, 82]). Notably, these observations are not limited to those with high BMI (overweight or obese), as similar relationships between higher BMI and lower parasympathetic activity have been shown in those with normal BMI [67]. Highlighting this close interplay between BMI and cardiovascular physiology, a study of adult obese and nonobese patients found that those who lost at least 10% of their body weight showed significant elevation in parasympathetic regulation and control as well as decreased sympathetic activation [5]. It has become increasingly clear, therefore, that cardiovascular autonomic function and physical well-being are tightly associated, with ANS function serving as a useful marker of health status and risk [61, 100].

Finally, to fully understand the influence of ANS on health, we must consider its function across the lifespan. A recent large, multi-cohort study of youth and young adults from age 6 months to 20 years was one of the first to comprehensively examine developmental trajectories of both the PNS and SNS from infancy to young adulthood [38]. The study found separate trajectories for the two branches. Specifically, parasympathetic function saw a steep increase from infancy to early childhood, followed by a plateau and then slight decline in late adolescence and early adulthood. Alternatively, the sympathetic branch was characterized by a gradual, linear decline across the developmental range [38]. Others have reported similar findings, including a steep increase in parasympathetic system activity early in development [2, 3, 108], followed by a plateau around middle childhood [24, 27, 39], and a relatively stable, linear decline in sympathetic activation throughout the childhood and adolescent years [2, 63]. Cardiovascular changes during the pubertal years are particularly noteworthy, as many hormones associated with pubertal onset and development may also influence autonomic control [21]. For example, in females, changes in HRV throughout the menstrual cycle have been reported, with a notable interaction between progesterone and HRV [89]. It is further hypothesized that changes in HRV across puberty are related to increased incidence of internalizing disorders [31] and clinical autonomic dysfunction [21], both of which show increased incidence during the adolescent developmental window (e.g., [21, 79, 109]). Thus, enhanced understanding of autonomic changes during pubertal maturation can likely provide insight into the link between cardiovascular physiology and overall health and well-being across the lifespan.

The above noted links between the ANS, physical health, and development highlight the need to study autonomic function and its dysfunction across a broad context of health and developmental factors. Especially as in some clinical populations, ANS differences may be more prevalent, with significant implications for overall health and well-being. In autism spectrum disorder (ASD), a neurodevelopment condition characterized by difficulties in social communication as well as restricted and repetitive behaviors [4], changes in autonomic functioning are frequently reported (see [8, 77]) for review). Several studies have reported reduced cardiovascular parasympathetic regulation in autistic children (e.g., [75, 78, 107]), adolescents (e.g., [25, 35, 70–72]), and adults [62, 93, 96]. Though fewer studies have investigated the sympathetic branch’s function in ASD, there is some evidence of atypical cardiovascular sympathetic activity in autistic individuals [26, 75]. The sum of these findings has led to proposals of a general state of hyperarousal in ASD [53], characterized by either reduced parasympathetic or elevated sympathetic states, which may predispose autistic individuals to a number of comorbidities associated with an unbalanced autonomic state, including but not limited to, cardiovascular disease [47, 101], poor emotional regulation [32], and internalizing disorders (e.g., [47, 51, 99]). However, it should be noted that others have reported no statistically significant differences in the ANS between those with and without ASD [54, 88, 110]. This heterogeneity becomes especially notable when accounting for other potential influencing factors, such as biological sex [14, 71], age [37, 70, 72], or medication use [97]. Further, minimal literature in ASD has addressed the balance or co-regulation of the two autonomic branches, instead focusing on one branch or the other in isolation. The few studies to date that have examined their interrelation have reported mixed results, with some reporting primarily differences in the PNS [75] while others have reported that the interaction of the two branches, rather than in isolation, is essential to understanding symptom profiles in ASD [69]. Clearly, a more in-depth analysis of autonomic functioning in both the PNS and SNS, with consideration of other influencing factors, is warranted to better understand cardiovascular autonomic balance and regulation in ASD.

In terms of physical health, autistic youth have been reported to have elevated BMI relative to neurotypical (NT) youth [12, 16, 29, 45, 64, 73, 87]. A study that examined the prevalence of obesity in adolescents using data from the 2017–2018 National Survey of Children’s Health found that the prevalence rate of obesity was significantly higher in autistic youth (25.1%) compared to NT youth (14.9%; [12]). In one study by Esteban-Figuerola and colleagues (2021), autistic youth with elevated BMI also had a higher waist circumference and waist/height ratio than NT youth. Prior research has shown that the odds of obesity increase with age in autistic youth from ages 10 to 17 [45, 73]. By adulthood, autistic individuals are likely at higher risk of a range of cardiovascular diseases [11, 22, 23, 40], further highlighting the need to understand autonomic dysfunction, and its relation to global health status, in ASD. Additionally, recent research suggests that ANS differences observed in ASD, such as reduced parasympathetic regulation, may be largely driven by elevated BMI [70, 72], raising questions as to whether diagnosis or other related individual factors might be driving reported dysregulation in ASD. Despite these findings, few studies in ASD have fully accounted for physical health status, particularly BMI, when examining diagnostic differences in ANS function, and no study, to our knowledge, has examined these effects over adolescent development.

Given the notable relationships and likely interplay between the ANS, pubertal development, and physical health, the objective of the current study was to examine resting-state cardiovascular autonomic functioning in autism in a large sample of children and adolescents, ranging in age from 10 to 13 years at enrollment and studied across four years for an age range spanning 10 to 17 years of age. The study aimed to identify change in autonomic function, as well as autonomic balance across both branches, over time as a function of diagnosis (ASD or NT), age, pubertal development, and physical health status. Specifically, the first aim was to examine the association of resting-state parasympathetic and sympathetic function with ASD diagnosis, age and pubertal development in youth with and without ASD accounting for the influence of average BMI and medication usage. We hypothesized that youth with ASD would have less parasympathetic regulation, which would remain stable and persist over development. Regarding sympathetic control, we expected the ASD group to show higher sympathetic activation. Further, we predicted that both average BMI and medication use would predict less PNS regulation and more SNS activation, regardless of diagnostic status or developmental stage. The second aim of the study sought to decompose the unique effect of BMI percentile at enrollment (Year 1) and within-participant change in BMI percentile over time on parasympathetic and sympathetic function, and autonomic balance/regulation, across the four-year study period. We expected Year 1 BMI percentile to predict lower PNS and higher SNS. Based on some literature showing changes in ANS profiles following weight loss [5], we hypothesized increase in BMI over time would be related to reduced PNS and elevated SNS activation.

## Methods

### Participants

Participants were enrolled at 10–13 years of age as part of a four-year longitudinal study of pubertal development in youth with and without ASD [15]. Participants were recruited from the southern United States covering a 200-mile radius that targeted medical and health-related services, clinics, research registries, regional disability organizations, schools, and social media platforms. Due to the task demands of the source study, all participants were required to have an intelligence quotient (IQ) ≥ 70 and spoken verbal fluency. The sample was not required to be medication-naïve. However, as part of the source longitudinal study’s criteria, any youth on medications that may alter endocrine function (e.g., corticosteroids, growth hormone, oral contraceptives) were excluded. Regarding medication status, in the ASD group 65.2% of were taking at least one medication compared to 17.5% in the NT group. Across the sample, medication use included stimulants, selective-serotonin reuptake inhibitors, melatonin, antihistamines, and central alpha-agonists. Those in the ASD group were required to have a clinical diagnosis of ASD, confirmed based on the Diagnostic and Statistical Manual-5 [4], and established by: (1) a diagnosis by a psychologist, psychiatrist, or behavioral pediatrician with autism expertise; (2) current clinical judgment; and (3) corroborated by the Autism Diagnostic Observation Schedule (ADOS-2; [56]), which was administered by research-reliable clinicians.

A total of 244 youth were enrolled, including 140 participants in the ASD group (36 females and 104 males) and 104 participants in the NT group (46 females and 58 males). In Y2 there were 183 participants, in Y3 there were 169 participants, and in Y4 there were 160 participants. The overall attrition rate was 33%, which was comparable to other longitudinal studies after the initial enrollment [74]. As demonstrated in previous reports on the study sample [17, 18], there was more attrition in the ASD group, often occurring after the Year 1 eligibility visit and diagnostic confirmation. Furthermore, the study team previously reported no difference in retained versus non-retained participants on key demographic variables (see [17]), and therefore, data were treated as missing at random. Years 2 and 3 study visits were impacted by the COVID-19 pandemic, when participants were unable to come in person due to stay-at-home restrictions. This resulted in missing data for primary autonomic outcomes for *N* = 43 youth at Y2 and *N* = 59 at Y3. Means and standard deviations of demographic and descriptive variables for the sample are provided in Table 1.

**Table 1:** Descriptive and Demographic Statistics Stratified by Diagnosis

	**NT**	**ASD**	***p*-value**
	**(*N* = 104)**	**(*N* = 140)**	
Age (Year 1)	11.71 (1.21)	11.43 (1.03)	0.058^1^
Age (Year 2)	12.86 (1.22)	12.63 (1.07)	0.168^1^
Age (Year 3)	13.87 (1.21)	13.59 (1.04)	0.115^1^
Age (Year 4)	14.74 (1.14)	14.59 (1.04)	0.375^1^
Sex			0.002^2^
Male	0.56 58/104	0.74 104/140	
Female	0.44 46/104	0.26 36/140	
Ethnicity			0.341^2^
Not Hispanic	0.95 99/104	0.92 129/140	
Hispanic	0.05 5/104	0.08 11/140	
Race			0.007^2^
Caucasian	0.86 89/104	0.81 114/140	
African American	0.02 2/104	0.12 17/140	
American Indian	0.00 0/104	0.00 0/140	
Asian/Pacific Islander	0.00 0/104	0.01 1/140	
Biracial	0.12 13/104	0.06 8/140	
Full-Scale IQ	117.22 (13.66)	101.33 (20.79)	< 0.001
ADOS-2 Comparison Score	7.21 (1.92)	–	–
SCQ-Lifetime (Year 1)	2.69 (2.49)	17.53 (8.35)	< 0.001
Psychotropic Medication (Year 1)	0.11 12/104	0.49 69/140	< 0.001^2^
BMI (Percentile, Year 1)	55.67 (31.23)	65.22 (31.16)	0.020^1^
Genital/Breast Development (Year 1)	1.97 (0.964)	2.05 (1.11)	0.559^1^
Height (Year 1)	59.80 (3.92)	59.36 (4.18)	0.415^1^
Weight (Year 1)	102.70 (34.61)	108.52 (41.06)	0.253^1^
Systolic BP (Year 1)	109.79 (11.96)	109.50 (10.81)	0.856^1^
Diastolic BP (Year 1)	63.44 (8.56)	64.24 (8.29)	0.508^1^
Heart Rate (Year 1)	75.33 (16.23)	76.02 (16.65)	0.775^1^
RSA (Year 1)	6.45 (0.80)	6.24 (0.99)	0.094^1^
PEP (Year 1)	95.21 (10.20)	94.25 (10.86)	0.543^1^

^1^Independent Sample T-Test, ^2^Pearson Chi-Square; Units: Height, In.; Weight, lb.; RSA, ln msec^2^; PEP, msec

Mean (Standard Deviation)

*BMI* Body Mass Index, *BP* Blood Pressure, *RSA* Respiratory Sinus Arrhythmia, *PEP* Pre-ejection Period

### Procedures

All study procedures were approved by the Vanderbilt Institutional Review Board and were carried out in accordance with the Code of Ethics of the World Medical Association (Declaration of Helsinki). Parents/guardians and youth participants provided written consent and written and verbal assent, respectively. Each year required one visit to the laboratory for completion of procedures.

***Autism Diagnostic Observation Schedule-Second Edition*** (ADOS-2; [56]) is a semi-structured interactive play and interview-based instrument used to support the diagnosis of ASD in conjunction with clinical judgement by a clinical psychologist, pediatrician, or psychiatrist with autism expertise. The ADOS-2 Module 3 was administered by research-reliable personnel. ADOS-2 Module 3 has good item reliability across items (88.2%), and good test–retest and interrater reliability (0.87). Furthermore, the ADOS-2 has good structural validity and demonstrates high sensitivity (91%) and specificity (84%).

***Social Communication Questionnaire*** (SCQ; [85]) is a screening tool to ascertain symptoms of ASD. The current study utilized the Lifetime version, which examines a child’s entire developmental history. The SCQ was administered to ASD and NT children, and a score of 15 or higher is suggestive of ASD. For the NT children, a score > 10 was exclusionary, but the threshold was not met by any participants. The SCQ has demonstrated good diagnostic sensitivity (0.71–0.78) and specificity (0.57 – 0.71) [19].

***Wechsler Abbreviated Scale of Intelligence, Second Edition*** (WASI-II, [111]) is a psychological measure of cognitive ability used to obtain an estimate of the participant’s intellectual functioning (IQ ≥ 70 required to participate in the study). Internal consistency in children is good to excellent (0.87 – 0.91) and concurrent validity with original, full-length Wechsler tests is acceptable to excellent (0.71 – 0.92) [111], which included ASD individuals [66].

#### Physical examination

Pubertal development was measured using physical exam based on Tanner stages at each annual visit according to standardized procedures [59, 60]. The exam obtained two measures with 5 stages from 1 (not begun) to 5 (fully developed) for Male External Genitalia (G1-G5 for males) and Female Breast (B1-B5 for females) (GB stage) and Pubic Hair (P1-P5 for both sexes) (PH stage). Visual inspection and categorization of pubertal and genital stage was completed without palpation of breasts or measurement of testes to be consistent with the original Tanner staging and maximize participation. Exams were conducted by trained, licensed male and female study physicians. At the beginning of each exam, the physicians spent roughly 5-min with the participant to establish rapport, explain the rationale for the exam and address any concerns. Subsequently, height and weight were obtained using a calibrated stand-on Health-o-meter TM Professional 499KL Waist High Digital Scale with Height Rod (Hogentogler & Co., MD, USA). Height was measured to the nearest inch and weight was measured to the nearest 0.1 lb*.* Children were weighed and measured in light clothing.

#### Body Mass Index (BMI)

BMI-for-age percentile was used to measure size and growth patterns as a proxy for the percentage of body fat. BMI has been shown to be a predictor of cardiovascular risk. BMI was calculated using the standard formula (lb/in^2^) × 703 for use with the Centers for Disease Control (CDC) growth charts for children and adolescents (2 through 19 years; https://www.cdc.gov/healthyweight/bmi/calculator.html). Percentiles and z-scores were calculated according to CDC growth charts based on sex and age. For the current study, BMI was considered as a proxy of physical health.

### Respiratory Sinus Arrhythmia (RSA) and Pre-ejection Period (PEP)

Cardiac autonomic measures were collected using MindWare Mobile Impedance Cardiograph units (MindWare Technologies LTD, Gahanna, OH), with synchronized measurement of electrocardiography (ECG), impedance cardiography, and respiration. Throughout the tasks, a highly trained research helper, who was either a full-time lab staff member or trained undergraduate research assistant, stayed with the participant to ensure comfort and adherence to protocols. Data collection began following a 35-min acclimation period to the lab environment and included a five-minute collection period in which participants were instructed to sit quietly without engaging in any other tasks. During the acclimation period, procedures for the study were reviewed with the participant, including placement of the electrodes. After reviewing the instructions and ensuring the child did not have any questions, the ECG electrodes were placed in a seven-electrode configuration for simultaneous measurement of ECG, impedance, and respiration. After, the 35-min acclimation period began in which participants were able to converse with the research helper or choose an activity from a collection of toys and games (e.g., coloring, activity book, cards, or board games), which were consistently provided to all participants. As described, the collection period consisted of a five-minute, sitting baseline, in which participants were instructed to sit quietly and were not allowed to engage in other tasks. The research helper was present during the collection to monitor participants. If the child wanted to engage or move around, the helper provided a scripted prompt to remind the child that “now, we are sitting quietly without talking.” Given the brevity of the task (five minutes), participants were consistently able to sit still and quietly for the duration of the baseline collection. Parasympathetic regulation was indexed using respiratory sinus arrhythmia (RSA), which is calculated as the variation in timing between successive heart beats in association with respiration. RSA was derived in accordance with the guidelines by the Society for Psychophysiological Research committee on heart rate variability [95] using the Heart Rate Variability Software Suite provided by MindWare Technologies (MindWare Technologies LTD, Gahanna, OH). Data was collected at 500 Hz sampling rate with respiration falling within the high frequency band (0.15 to 0.42 Hz) in one-minute epochs. Sympathetic control was measured as pre-ejection period (PEP), the interval from electrical stimulation to mechanical opening of the aorta and calculated as the distance (in ms) from the ECG Q-point of the QRS complex to the B point of the impedance waveform, which corresponds with the time from ventricular depolarization to aortic valve opening [90]. Higher PEP indicates a longer time from depolarization to valve opening, and therefore, less sympathetic control. PEP was ensemble-averaged for each one-minute epoch and B-point calculated at 55% of the R-Z interval (time to dZ/dt peak) [57] in the Impedance Cardiography Software Suite (MindWare Technologies LTD, Gahanna, OH). The software suites include automatic R-peak detection algorithms, which automatically detect and label R-peaks based on waveform slope, peak sharpness, and amplitude of the peak relative to the rest of the waveform [68]. Both the QRS complex and dZ/dt signal were confirmed by visual inspection by the lead author (RAM) or trained research assistants to identify incorrectly labeled R-peaks due to motion or arrhythmia. Research assistants had to reach an inter-rater reliability of kappa = 0.80 before working with experimental data, and any instances of disagreement between the lead author and assistant were discussed and a consensus decision reached. If greater than 5% of the total peaks in a one-minute epoch required manual correction, that minute was excluded from analyses. For RSA, a total 2.8% of data were excluded due to excessive motion artifact, arrhythmia, or equipment error. For impedance cardiography and PEP, which is more sensitive to motion artifact, a total 7.8% of data were excluded.

CAB and CAR were calculated using procedures first described in [10]. In short, RSA and PEP values were normalized as z-scores (zRSA and zPEP), and because lower PEP scores indicate more sympathetic control, these values were multiplied by −1 to create a positive association (-zPEP). CAB, the difference between parasympathetic and sympathetic control, was calculated as zRSA – (-zPEP), with higher values indicating more parasympathetic dominance. CAR, an overall sum of autonomic control, was calculated as zRSA + (-zPEP), where higher scores indicate more overall autonomic regulation [10].

### Statistical analysis

Hypotheses were tested within linear mixed effects models with a random intercept for participant to account for correlation within participants using Satterthwaite degrees of freedom. The first aim sought to examine the association of each of the four primary outcomes, RSA, PEP, CAB, and CAR, with development (age or GB stage) and diagnosis using four linear mixed models, controlling for sex. To investigate the impact of controlling for BMI and medication usage on the development and diagnosis associations, we fit all models with BMI percentile and psychotropic medication use, then subsequently fit models to control for known differences in these variables between diagnostic groups. The second aim of the study examined the extent to which BMI percentile at enrollment (Year 1) and the within-participant change in BMI percentile over time influenced an individual’s change in autonomic function across the four-year study period. These models built on those from the first aim and included main effects for diagnosis, biological sex, age (or GB stage), BMI percentile at enrollment (Y1 BMI), ΔBMI, year, and psychotropic medication use. Effect sizes for longitudinal mixed models were calculated as Cohen’s f^2^, with 0.02, 0.15, and 0.35 representing small, medium, and large effects, respectively. Effect sizes for model estimates (t-tests) were calculated as Cohen’s d (0.2 = small effect, 0.5 = medium effect, 0.8 = large effect) (Supplement of [46, 106]). All analyses were performed using IBM SPSS Statistics 29 [41].

## Results

### Aim 1: Effects of age and pubertal development

#### Baseline models

First, diagnostic effects were examined using baseline models with diagnosis, biological sex, and age (or GB stage). ASD diagnosis was significantly associated with lower RSA (less parasympathetic regulation; Table 2). Diagnosis was not statistically significant for PEP, CAR, or CAB (Table 2).

**Table 2:** Type III Sum of Squares ANOVA Table for Age, Diagnosis, and Sex on Autonomic Outcomes

	**F**	**df**	**p-value**	***f***^**2**^
**Respiratory Sinus Arrhythmia (RSA)**				
Diagnosis	5.848	1	**0.016**	**0.023**
Sex	0.411	1	0.522	0.000
Age	0.090	1	0.764	0.000
**Pre-ejection Period (PEP)**				
Diagnosis	0.068	1	0.794	0.000
Sex	0.702	1	0.403	0.000
Age	204.831	1	** < 0.001**	**0.966**
**Cardiac Autonomic Balance (CAB)**				
Diagnosis	2.422	1	0.121	0.007
Sex	0.253	1	0.616	0.000
Age	2.347	1	0.126	0.006
**Cardiac Autonomic Regulation (CAR)**				
Diagnosis	1.951	1	0.164	0.004
Sex	1.076	1	0.301	0.000
Age	2.922	1	0.088	0.009

Bold indicates significance at p < 0.05

#### RSA

**Fig. 1:**
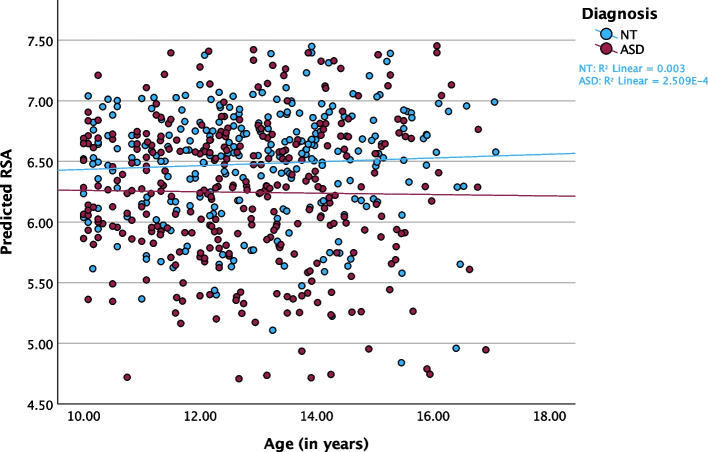
Predicted RSA by Age in ASD and NT Youth. Scatterplot of predicted RSA values over the age trajectory, stratified by diagnosis and controlling for linear model fixed and random effects

Medication use and BMI percentile were added to the baseline model to control for potential differences in these variables between groups. After adding these variables, the main effect for diagnosis was not statistically significant (F_(1, 234.344)_ = 0.245, *p* = 0.621, f^2^ = 0.00). Medication use was significantly associated with lower RSA values (F_(1, 511.417)_ = 12.540, *p* < 0.001, f^2^ = 0.055), indicating less parasympathetic regulation with use of psychotropic medication (Table 3). Similarly, higher BMI was significantly associated with lower RSA (*p* < 0.001; Table 3). The main effect for age was not statistically significant (F_(1, 551.288)_ = 0.090, *p* = 0.765, f^2^ = 0.00), suggesting that RSA levels were stable over adolescence (Fig. 1). In models with GB stage rather than age, results were similar, and as with age, RSA did not significantly differ over pubertal development stage (*p* = 0.119; Additional File 1, Tables S1 and S2).

**Table 3:** Aim 1: Model Estimates for Age, Diagnosis, Sex, BMI Percentile, and Medication on Autonomic Outcomes

Parameter	Estimate	rdf	95% CI	t	*p*-value	d
**RSA**						
Intercept	6.757	566	6.155, 7.358	22.069	< 0.001	3.046
Diagnosis: ASD	−0.057	234.344	−0.282, 0.169	−0.495	0.621	−0.068
Sex: Female	−0.071	214.275	−0.293, 0.150	−0.635	0.526	−0.088
Medication Use: Yes	−0.339	511.417	−0.527, −0.151	−3.541	< 0.001	−0.489
Age	0.007	551.288	−0.037, 0.050	0.3	0.765	0.041
BMI Percentile	−0.005	368.836	−0.008, −0.002	−3.386	< 0.001	−0.467
σ^2^_Error_	0.507					
σ^2^_Participant_	0.350					
**PEP**						
Intercept	58.499	498.302	52.352, 64.647	18.696	< 0.001	2.580
Diagnosis: ASD	0.55	219.240	−2.288, 3.388	0.382	0.703	0.053
Sex: Female	0.569	195.277	−2.257, 3.394	0.397	0.692	0.055
Medication Use: Yes	−0.158	511.897	−2.165, 1.849	−0.154	0.877	−0.021
Age	3.091	430.411	2.662, 3.519	14.181	< 0.001	1.957
BMI Percentile	0.011	423.644	−0.024, 0.045	0.609	0.543	0.084
σ^2^_Error_	38.131					
σ^2^_Participant_	69.705					
**CAB**						
Intercept	−0.427	510	−1.340, 0.485	−0.919	0.358	−0.127
Diagnosis: ASD	−0.039	230.820	−0.416, 0.337	−0.206	0.837	−0.028
Sex: Female	0.039	206.248	−0.332, 0.410	0.207	0.837	0.029
Medication Use: Yes	−0.346	510	−0.639, −0.053	−2.318	0.021	−0.320
Age	0.066	467.840	0.001, 0.131	2.01	0.045	0.277
BMI Percentile	−0.004	381.706	−0.009, 0.00	−1.801	0.073	−0.249
σ^2^_Error_	0.934					
σ^2^_Participant_	1.080					
**CAR**						
Intercept	1.093	500.742	0.266, 1.919	2.598	0.01	0.359
Diagnosis: ASD	−0.021	216.858	−0.367, 0.326	−0.118	0.906	−0.016
Sex: Female	−0.115	193.288	−0.457, 0.227	−0.661	0.509	−0.091
Medication Use: Yes	−0.365	500.164	−0.631, −0.099	−2.691	0.007	−0.371
Age	−0.042	454.839	−0.101, 0.017	−1.406	0.16	−0.194
BMI Percentile	−0.006	371.400	−0.010, −0.002	−2.635	0.009	−0.364
σ^2^_Error_	0.753					
σ^2^_Participant_	0.934					

Bold indicates significance at *p* < 0.05. rdf = residual degrees of freedom; CI = Confidence Interval; d = Cohen’s d; σ^2^_Error_ = Error Variance; σ^2^_Participant_ = Random Effects Variance

#### PEP

**Fig. 2:**
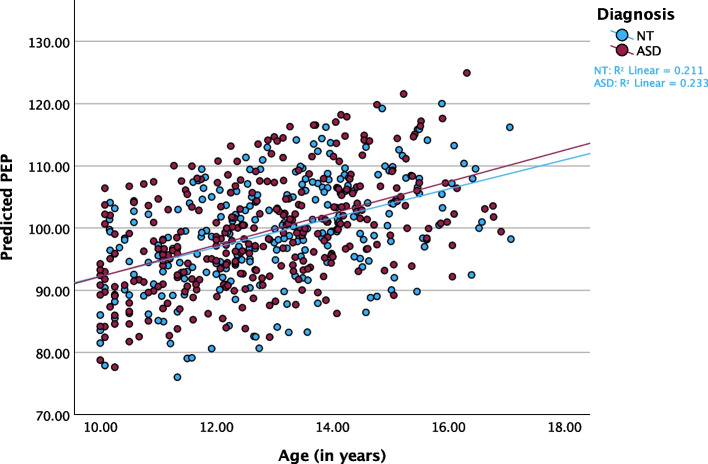
Predicted PEP by Age in ASD and NT Youth. Scatterplot of predicted PEP values over the age trajectory, stratified by diagnosis and controlling for linear model fixed and random effects

Similarly to RSA, there was no statistically significant effect of diagnosis (F_(1, 219.240)_ = 0.146, *p* = 0.703, f^2^ = 0.00). However, PEP increased with age (*p* < 0.001), suggesting that basal sympathetic control decreases as youth age, regardless of diagnostic status (Table 3; Fig. 2). Neither BMI percentile (F_(1, 423.644)_ = 0.371, *p* = 0.543, f^2^ = 0.00) nor medication use (F_(1, 511.897)_ = 0.024, *p* = 0.877, f^2^ = 0.00) were significantly associated with PEP. Regarding GB stage, model results mirrored findings from those with age, with pubertal maturation significantly associated with increased PEP (reduced sympathetic control; *p* < 0.001; Additional File 1, Table S1 and S2).

### Cardiac autonomic balance and reactivity

**Fig. 3:**
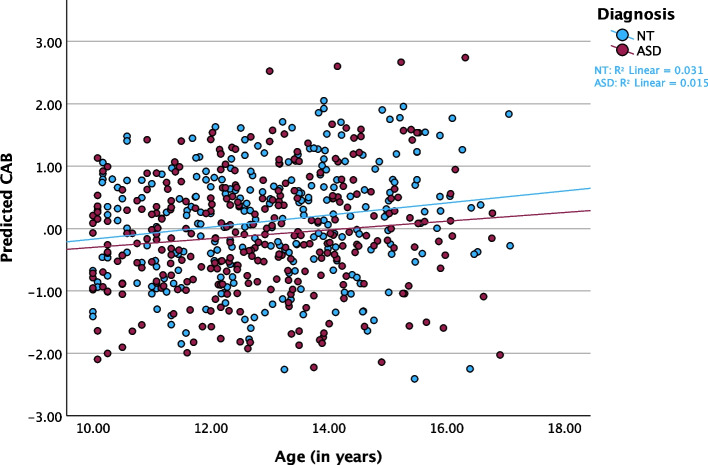
Predicted CAB by Age in ASD and NT Youth. Scatterplot of predicted CAB values over the age trajectory, stratified by diagnosis and controlling for linear model fixed and random effects

**Fig. 4:**
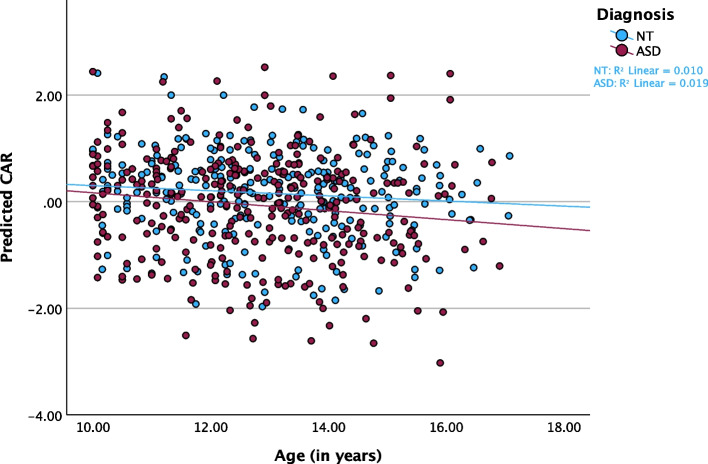
Predicted CAR by Age in ASD and NT Youth. Scatterplot of predicted CAR values over the age trajectory, stratified by diagnosis and controlling for linear model fixed and random effects

For CAB, both age (F_(1,467.840)_ = 4.041, *p* = 0.045, f^2^ = 0.014) and GB stage (F_(1, 485.022)_ = 6.927, *p* = 0.009, f^2^ = 0.028) were statistically significant. However, there was no statistically significant effect for diagnosis in CAB (F_(1,230.820)_ = 0.042, *p* = 0.837, f^2^ = 0.000) or CAR (F_(1,216.858)_ = 0.014, *p* = 0.906, f^2^ = 0.000). In both cases, increasing age (Fig. 3; Table 3) or physical development (Additional File 1, Table S2) was associated with an increase in CAB, which suggests a transition to greater parasympathetic control (relative to sympathetic) over development. In contrast, for CAR or total autonomic reactivity neither main effects for age (F_(1, 454.839)_ = 1.978, *p* = 0.160, f^2^ = 0.005; Fig. 4) nor for GB stage (F_(1, 480.580)_ = 0.720, *p* = 0.397, f^2^ = 0.00) were statistically significant.

## Aim 2: Effects of baseline BMI and BMI change

### RSA

Models separately fitting baseline BMI and change from baseline were used to investigate between- and within-participant associations of BMI with autonomic functioning. Results for non-BMI variables, including diagnosis and age/pubertal stage were consistent with Aim 1 (see Table 4, Table S3, and S4). Y1 BMI was a statistically significant predictor of RSA (F_(1, 218.885)_ = 11.056, *p* = 0.001, f^2^ = 0.047). Thus, individuals with higher BMI at Y1 had lower RSA values on average (Table 4). The effect for ΔBMI was not statistically significant (F_(1, 552.010)_ = 2.12, *p* = 0.146, f^2^ = 0.005). Results were similar when controlling for GB stage rather than age, although advancing GB stage was significantly associated with a slight increase in RSA (*p* = 0.010; Additional File 1, Tables S3 and S4).

**Table 4:** Aim 2: Model Estimates for Age, Diagnosis, Sex, Medication, BMI at Y1, and Change in BMI on Autonomic Outcomes

Parameter	Estimate	rdf	95% CI	t	p-value	d
**RSA**						
Intercept	6.371	227.780	5.368, 7.374	12.514	< 0.001	1.727
Diagnosis: ASD	−0.048	231.515	−0.274, 0.179	−0.416	0.678	−0.057
Sex: Female	−0.074	214.955	−0.297, 0.149	−0.654	0.514	−0.090
Medication Use: Yes	−0.334	516.189	−0.523, −0.145	−3.476	< 0.001	−0.480
Time	−0.058	323.718	−0.168, 0.053	−1.029	0.304	−0.142
Age	0.049	221.661	−0.043, 0.140	1.042	0.298	0.144
Y1 BMI Percentile	−0.006	218.885	−0.009, −0.002	−3.325	0.001	−0.459
BMI Change	−0.004	552.010	−0.009, 0.001	−1.456	0.146	−0.201
σ^2^_Error_	0.506					
σ^2^_Participant_	0.354					
**PEP**						
Intercept	70.603	209.661	58.163, 83.044	11.188	< 0.001	1.544
Diagnosis: ASD	0.203	212.170	−2.619, 30.25	0.142	0.887	0.020
Sex: Female	0.623	193.728	−2.172, 3.417	0.439	0.661	0.061
Medication Use: Yes	−0.267	509.991	−2.271, 1.737	−0.262	0.794	−0.036
Time	1.52	263.965	0.208, 2.832	2.281	0.023	0.315
Age	1.86	207.229	0.717, 3.003	3.207	0.002	0.443
Y1 BMI Percentile	0.019	196.264	−0.023, 0.062	0.906	0.366	0.125
BMI Change	−0.003	419.181	−0.054, 0.048	−0.116	0.908	−0.016
σ^2^_Error_	38.335					
σ^2^_Participant_	67.341					
**CAB**						
Intercept	−1.576	219.689	−3.235, 0.083	−1.872	0.063	−0.258
Diagnosis: ASD	−0.02	226.471	−0.397, 0.357	−0.103	0.918	−0.014
Sex: Female	0.026	206.319	−0.344, 0.397	0.14	0.888	0.019
Medication Use: Yes	−0.333	508	−0.626, −0.039	−2.227	0.026	−0.307
Time	−0.147	293.334	−0.326, 0.032	−1.617	0.107	−0.223
Age	0.18	215.275	0.027, 0.332	2.325	0.021	0.321
Y1 BMI Percentile	−0.004	209.444	−0.010, 0.001	−1.488	0.138	−0.205
BMI Change	−0.005	459.063	−0.013, 0.003	−1.243	0.215	−0.172
σ^2^_Error_	0.936					
σ^2^_Participant_	1.069					
**CAR**						
Intercept	1.989	203.626	0.464, 3.514	2.572	0.011	0.355
Diagnosis: ASD	−0.018	209.618	−0.365, 0.328	−0.103	0.918	−0.014
Sex: Female	−0.094	190.720	−0.435, 0.247	−0.543	0.588	−0.075
Medication Use: Yes	−0.374	497.238	−0.641, −0.108	−2.762	0.006	−0.381
Time	0.099	270.722	−0.013, −0.003	1.196	0.233	0.165
Age	−0.12	199.751	−0.260, 0.20	−1.691	0.092	−0.233
Y1 BMI Percentile	−0.008	193.561	−0.013, −0.003	−3.079	0.002	−0.425
BMI Change	−0.002	443.460	−0.009, 0.005	−0.505	0.614	−0.070
σ^2^_Error_	0.753					
σ^2^_Participant_	0.921					

Bold indicates significance at *p* < 0.05. rdf = residual degrees of freedom; CI = Confidence Interval; d = Cohen’s d; σ^2^_Error_ = Error Variance; σ^2^_Participant_ = Random Effects Variance

## PEP

There was evidence of a statistically significant increase in PEP (decrease in sympathetic control) over the four-year period (F_(1, 263.965)_ = 5.202, *p* = 0.023, f^2^ = 0.020). However, main effects of Y1 BMI (F_(1, 196.264)_ = 0.821, *p* = 0.366, f^2^ = 0.00) and ΔBMI (F_(1, 419.181)_ = 0.014, *p* = 0.908, f^2^ = 0.00) were not statistically significant. When controlling for GB Stage instead of age, statistically significant model results did not differ (see Additional File 1, Tables S3 and S4).

### Cardiac autonomic balance and reactivity

In models with CAB as the outcome, neither Y1 BMI (F_(1, 209.444)_ = 2.215, *p* = 0.138, f^2^ = 0.006) nor ΔBMI (F_(1, 459.063)_ = 1.545, *p* = 0.215, f^2^ = 0.003) were statistically significant. For CAR, larger Y1 BMI percentile was associated with lower CAR in models with age (*p* = 0.002; Table 4) or GB stage (*p* = 0.004; Additional File 1, Table S4).

## Discussion

The role of the ANS in ASD continues to be of great interest to researchers in the field. With a theorized link between autonomic function, particularly within the parasympathetic system, and social behavior (e.g., [80]), a growing number of studies have explored whether markers of the ANS, such as heart rate variability, may also serve as a diagnostic marker for ASD. Indeed, many studies report differences in the ANS in ASD (see [8, 14, 28] for review),however, the extent to which these differences are unique to ASD remains under investigated. Furthermore, little is known about the trajectory of ANS function (or dysfunction) over time in this population, including during the adolescent period, a time of considerable physical, psychological, and physiological change. The current study is one of the first to our knowledge to explicitly examine the longitudinal progression of heart rate markers of parasympathetic and sympathetic function in school-age and adolescent youth with and without ASD. It was predicted that youth with ASD would have less PNS regulation and more SNS activation over development, which would be significantly related to medication usage, BMI, and change in BMI over time. Study of this large, well-characterized sample found that while there were diagnostic differences in the PNS in ASD when other confounds were not considered, these differences may more likely be the result of co-occurring symptoms in ASD, rather than specific to an ASD diagnosis itself.

The first aim was to examine ANS function without accounting for BMI and medication usage. In these baseline models, we found the expected diagnostic effect where individuals with ASD demonstrated lower RSA. However, groups did not differ in the sympathetic branch (PEP), nor did they differ in terms of balance (CAB) or reactivity (CAR). These findings are largely consistent with the extant literature, with several previous studies reporting lower basal RSA in youth with ASD [25, 35, 62, 70–72, 75, 78, 93, 96, 107]. Although fewer studies have investigated basal sympathetic function or autonomic balance, most that have done so have not identified statistically significant diagnostic effects at rest [26, 69, 88].

Additionally, despite the growing interest in studying the ANS in ASD, very little research has investigated these measures longitudinally. However, it is important to identify whether there are diagnostic differences in autonomic function during key developmental windows, such as adolescence and puberty. It is not clear whether differences in autonomic function worsen, remain stable, or improve as autistic individuals age. Further, the ANS has been linked to many physical (e.g., [101]) and mental health (e.g., [7, 51]) conditions, some of which are first diagnosed or increase in prevalence during adolescence (e.g., [21, 79]). The rates of these conditions are even higher in ASD [22, 34, 92, 104]. Therefore, understanding the relationship between ASD and the ANS during pubertal development will likely expand insight into how autonomic health may be associated with, predict, or even worsen co-occurring conditions often observed in ASD during developmental progression. While it was hypothesized that the ASD youth would show a different developmental trajectory over age and pubertal development, this was not supported. Instead, both groups demonstrated relatively stable RSA over the adolescent window and across developmental stages, which is consistent with literature in non-autistic populations [24, 27, 39]. Meanwhile, both groups showed an increase in PEP over age and puberty, which marked a decrease in sympathetic control over time, again consistent with research in non-autistic youth [2, 38, 63]. Finally, autonomic balance (CAB) increased over time, while overall regulation (CAR) remained stable. In considering these findings together, it is apparent that as youth in both diagnostic groups age and develop, a more parasympathetic-dominant profile emerges. These findings are encouraging, particularly in autistic youth, as this shift towards parasympathetic dominance and less sympathetic control is considered highly adaptive and a key marker of overall health and well-being [100, 101], including for both improved cardiovascular health [101] and psychological well-being and emotion regulation [32, 98].

Several studies have highlighted relationships between individual characteristics and the ANS. For example, there is evidence that traits such as emotion regulation [6], attention [33], executive function [58], biological sex [38, 52], and internalizing characteristics (e.g., [13, 51]) are all influenced by or influence autonomic physiology. This diverse array of individual traits emphasizes the limitations of studying the ANS under a categorical lens, leading many to advocate for a more dimensional, trait-based approach [7]. Similarly, results from the current study found that inclusion of two notable health variables – psychotropic medication use and BMI percentile – resulted in a lack of significance for the observed diagnostic effect in the baseline models. The finding that inclusion of confounding variables known to independently influence ANS function negates any observed diagnostic effects mirrors previous findings by our group, where we reported that youth with ASD vs NT did not differ in RSA during a stressor after inclusion of BMI percentile [70, 72] and provides additional evidence to the value of a dimensional approach.

Regarding medication use, a recent study reported that approximately half of all autistic children on Medicaid were prescribed at least one psychotropic in a given year [83] while a review of studies published from 1976 to 2012 identified prevalence ranges of anywhere from 2.7% to 80% [44]. Further, reviews of the extant literature have found that these psychotropic medications can influence autonomic function (e.g., [47, 48]). For example, antidepressants, and particularly tricyclic antidepressants (e.g., [105]), have been shown to reduce HRV through anticholinergic properties [43], though the impact of selective serotonin reuptake inhibitors (SSRIs) on HRV is less clear and may be more benign (e.g., [48, 86]). Similarly, a meta-analytic review of stimulant and non-stimulant medications in individuals with attention-deficit/hyperactivity disorder showed significant reduction in HRV with medication usage [42]. Findings from the current study were similar, with medication usage being associated with significantly less parasympathetic regulation and reduced overall cardiac autonomic regulation. However, we did not find a statistically significant effect for medications specifically for the SNS or CAB. One reason for this discrepancy may be that medications that are known to affect sympathetic function direction through beta-adrenergic blockade (e.g., propranolol, atenolol) [94] are not as commonly prescribed in ASD, with no participants on a beta-blocker in this study. Additionally, participants were on a broad range of psychotropics, and many were on more than one (see Additional File 2). The mechanisms of action for typical stimulants (e.g., methylphenidate) versus serotonin reuptake inhibitors (e.g., fluoxetine) versus alpha-agonists (e.g., guanfacine) all differ, and as a result, influence cardiovascular function differently and in some cases, in opposite directions. Clearly, a more in-depth, detailed examination of types and combinations of medications used will be necessary to more completely elucidate the extent to which pharmacological mechanisms may explain differences in autonomic function in ASD.

As with medication use, BMI percentile was highly correlated with reduced parasympathetic regulation and less overall autonomic regulation. Prior research has shown that autistic youth often evidence elevated BMI compared to NT youth [12, 16, 29, 45, 64, 73, 87]. Similarly, a significantly higher prevalence of obesity in autistic compared to NT adolescents has been found [12] with significant increases between 10 to 17 years [45, 73] which parallels the age range of the current study.

Interestingly, in Aim 2, when separating BMI percentile into two variables – BMI percentile at enrollment and change in BMI across the four-year study period – only BMI at enrollment was statistically significant. This is in contrast with clinical studies in adult populations that reported immediate, notable changes in HRV following change in weight (e.g., weight loss) [5]. It will be necessary to further examine this relationship in future research, especially considering the associations between BMI, HRV, and overall heart health. In depth, large scale longitudinal studies across a broad age range will be informative in identifying the extent to which body weight and body mass directly contribute to changes in autonomic health, as well as the potential to reverse such changes with lifestyle modifications.

However, it is valid to note that BMI is not a perfect index of physical health or even body fat [84]. Recently, other measures that may be more accurate in clearly defining an individual’s amount of body fat (e.g., waist-circumference-to-height ratio), have gained more influence within the medical community as an improved alternative to BMI [1]. In has been shown that autistic youth with elevated BMI also had a higher waist circumference and waist/height ratio than NT youth [29]. Nevertheless, previous reports of elevated BMI in autistic individuals both by the study team [16] and others [12, 29, 45, 64, 73, 87] emphasize the need to better understand how changes in weight or overall health status might be driving some of the reported differences in autonomic function in this population. At a minimum, findings highlight the need to control for these confounding variables in studies seeking to identify whether the ANS or HRV metrics can be used as biological markers of ASD or autistic traits.

## Limitations and future directions

The current study has several notable strengths. First, the study examined a relatively large sample of youth with and without ASD that were well-characterized clinically as part of the source longitudinal study [15]. The examination of both branches of the ANS – parasympathetic and sympathetic – was a strength, as little research in ASD has examined them simultaneously, nor have studies looked at these measures longitudinally across several years in an autistic population. Despite these strengths, there are several key limitations to mention. Firstly, the later years of the study were conducted during the COVID-19 pandemic, and therefore, there was missing autonomic data for some participants, primarily in Y2 and Y3, due to having to implement virtual study visits. Due to the cognitive demands of the parent study [15], participants were required to have an IQ of at least 70, limiting the generalizability of the findings to the broader autism spectrum. The sample was primarily White, and future research should focus on including a more cognitively, racially, and ethnically diverse sample. Additionally, autistic females were underrepresented in the sample, likely hindering our ability to detect possible sex-based differences. Efforts are currently underway to recruit and follow a large sample of autistic and neurotypical females to allow for the necessary elucidation of sex-based association with HRV, physical development, and psychological well-being. The study was limited in its use of physical indicators of health, relying primarily upon medication usage and BMI percentile. As acknowledged, BMI is an imperfect measure and not a complete proxy of physical health, and future research will need to more completely examine not only body composition, but other potentially important variables such as activity level or blood-based markers of physical well-being (e.g., cholesterol). The study was limited to examining a five-minute baseline resting period, which provides merely a snapshot of an individual’s overall autonomic function. There is great need in the field to study and examine ANS function over an extended period of time, such as 24-h measures of cardiovascular variability. We did not quantify clinical comorbidities beyond ASD, nor were other co-occurring characteristics, such as sensory sensitivities, language, emotional regulation, or adaptive behaviors included in analyses. Moving forward, efforts should be made to look at physical and psychological characteristics in relation to ANS function, especially in considering the potential psychological impact of differences in PNS and SNS balance (e.g., [98]). Finally, the study examined medication usage using a broad psychotropic medication category. Future studies in larger samples will allow for sufficient power to assess more specific medication classes (e.g., stimulants, SSRIs, etc.) in relation to ANS control.

## Conclusions

While considerable research has been conducted examining the core symptoms and mental health consequences of an ASD diagnosis, less investment has been spent on elucidating the physical health outcomes in ASD. The current study demonstrates the importance of examining this under-researched area, informing our understanding of the relationship between physical and autonomic health in ASD. The findings in Aim 1, with a lack of statistically significant diagnostic effect when accounting for BMI and medication use, suggest future research may benefit from a shift in diagnostic-based comparisons to a trait-based approach. This study is not the first to recommend a focus on traits rather than diagnostic category. Beauchaine and Thayer [7] proposed HRV as a *transdiagnostic* marker of psychopathology and advocated for a dimensional approach focused on characteristics rather diagnosis. In the current study, very few group differences were observed at the diagnostic level, especially when including important confounding variables. Instead, future research may be more fruitful with a focus on differences at an individual level and an aim towards examining the extent to which autonomic dysfunction or differences are related to specific, unique clinical phenotypes. Specifically, we aim to examine an individual’s expression of characteristics often implicated in or co-occurring in ASD, such as social difficulties, internalizing symptoms, or medication usage as the focal point of comparison, rather than a categorical grouping based upon diagnosis. An enhanced understanding of the ANS in ASD has the potential to offer insights into both mental and physical health status, with critical implications for identifying characteristics that may influence overall well-being throughout the lifespan.

## Supplementary Information

Additional file 1: Supplementary Tables 1-4. Table S1. Fixed Effects for Aim 1 with GB Stage; Table S2. Model Estimates for Aim 1 with GB Stage; Table S3. Fixed Effects for Aim 2 with GB Stage; Table S4. Model Estimates for Aim 2 with GB Stage

Additional file 2: Overview of Psychotropic Medication Use by Diagnostic Group and Year

## Data Availability

Participant demographic and outcome variables are available from the NIMH Data Archive Collection 2683. The physiological datasets used and analyzed are available from the corresponding author upon reasonable request.

## Abbreviations

ASD: Autism Spectrum Disorder
ANS: Autonomic Nervous System
BMI: Body Mass Index
CAB: Cardiac Autonomic Balance
CAR: Cardiac Autonomic Regulation
GB: Genital/Breast Stage
HRV: Heart Rate Variability
NT: Neurotypical
PNS: Parasympathetic Nervous System
PEP: Pre-ejection Period
RSA: Respiratory Sinus Arrhythmia
SNS: Sympathetic Nervous System

## References

[1] Agbaje AO. Waist-circumference-to-height-ratio had better longitudinal agreement with DEXA-measured fat mass than BMI in 7237 children. Pediatr Res. 2024;96(5):1369–80. 10.1038/s41390-024-03112-8. 38443520 10.1038/s41390-024-03112-8 PMC11522001

[2] Alkon A, Boyce WT, Davis NV, Eskenazi B. Developmental changes in autonomic nervous system resting and reactivity measures in Latino children from 6 to 60 months of age. J Dev Behav Pediatr. 2011;32(9):668–77. 10.1097/DBP.0b013e3182331fa6. 22008788 10.1097/DBP.0b013e3182331fa6

[3] Alkon A, Goldstein LH, Smider N, Essex MJ, Kupfer DJ, Boyce WT. Developmental and contextual influences on autonomic reactivity in young children. Dev Psychobiol. 2003;42(1):64–78. 10.1002/dev.10082. 12471637 10.1002/dev.10082

[4] American Psychiatric Association. (2013). Diagnostic and Statistical Manual of Mental Disorders, 5th Edition: DSM-5 (Fifth Edition). American Psychiatric Publishing. 10.1176/appi.books.9780890425596

[5] Arone LJ, Mackintosh R, Rosenbaum M, Leibel RL, Hirsch J. Autonomic nervous system activity in weight gain and weight loss. Am J Physiol. 1995;269(1):R222-225. 10.1152/ajpregu.1995.269.1.R222. 7631897 10.1152/ajpregu.1995.269.1.R222

[6] Beauchaine TP. Respiratory Sinus Arrhythmia: A Transdiagnostic Biomarker of Emotion Dysregulation and Psychopathology. Curr Opin Psychol. 2015;3:43–7. 10.1016/j.copsyc.2015.01.017. 25866835 10.1016/j.copsyc.2015.01.017 PMC4389219

[7] Beauchaine TP, Thayer JF. Heart rate variability as a transdiagnostic biomarker of psychopathology. Int J Psychophysiol. 2015;98(2 Pt 2):338–50. 10.1016/j.ijpsycho.2015.08.004. 26272488 10.1016/j.ijpsycho.2015.08.004

[8] Benevides TW, Lane SJ. A review of cardiac autonomic measures: Considerations for examination of physiological response in children with autism spectrum disorder. J Autism Dev Disord. 2015;45(2):560–75. 10.1007/s10803-013-1971-z. 24154761 10.1007/s10803-013-1971-z

[9] Berntson GG, Cacioppo JT, Quigley KS. Cardiac psychophysiology and autonomic space in humans: empirical perspectives and conceptual implications. Psychol Bull. 1993;114(2):296–322. 10.1037/0033-2909.114.2.296. 8416034 10.1037/0033-2909.114.2.296

[10] Berntson GG, Norman GJ, Hawkley LC, Cacioppo JT. Cardiac autonomic balance versus cardiac regulatory capacity. Psychophysiology. 2008;45(4):643–52. 10.1111/j.1469-8986.2008.00652.x. 18282204 10.1111/j.1469-8986.2008.00652.x PMC3767155

[11] Bishop-Fitzpatrick L, Movaghar A, Greenberg JS, Page D, DaWalt LS, Brilliant MH, Mailick MR. Using machine learning to identify patterns of lifetime health problems in decedents with autism spectrum disorder. Autism Res. 2018;11(8):1120–8. 10.1002/aur.1960. 29734508 10.1002/aur.1960 PMC6203659

[12] Buro AW, Salinas-Miranda A, Marshall J, Gray HL, Kirby RS. Obesity and neurodevelopmental and mental health conditions among adolescents aged 10–17 years: The National Survey of Children’s Health 2017–2018. J Paediatr Child Health. 2022;58(10):1753–9. 10.1111/jpc.16081. 35748345 10.1111/jpc.16081 PMC10165505

[13] Chalmers, J. A., Quintana, D. S., Abbot, M. J.-A., & Kemp, A. H. (2014). Anxiety disorders are associated with reduced heart rate variability: a meta-analysis. Frontiers in Psychiatry, 5. 10.3389/fpsyt.2014.00080 10.3389/fpsyt.2014.00080 PMC4092363 25071612

[14] Cheng YC, Huang YC, Huang WL. Heart rate variability in individuals with autism spectrum disorders: A meta-analysis. Neurosci Biobehav Rev. 2020;118:463–71. 10.1016/j.neubiorev.2020.08.007. 32818581 10.1016/j.neubiorev.2020.08.007

[15] Corbett, B. A. (2017). Examining stress and arousal across pubertal development in ASD (R01 MH111599) [Grant].

[16] Corbett BA, Muscatello RA, Horrocks BK, Klemencic ME, Tanguturi Y. Differences in Body Mass Index (BMI) in Early Adolescents with Autism Spectrum Disorder Compared to Youth with Typical Development. J Autism Dev Disord. 2021;51(8):2790–9. 10.1007/s10803-020-04749-0. 33051783 10.1007/s10803-020-04749-0 PMC8041918

[17] Corbett BA, Muscatello RA, Kim A, Vandekar S, Duffus S, Sparks S, Tanguturi Y. Examination of pubertal timing and tempo in females and males with autism spectrum disorder compared to typically developing youth. Autism Res. 2022;15(10):1894–908. 10.1002/aur.2786. 35912944 10.1002/aur.2786 PMC9561009

[18] Corbett BA, Muscatello RA, McGonigle T, Vandekar S, Burroughs C, Sparks S. Trajectory of depressive symptoms over adolescence in autistic and neurotypical youth. Molecular Autism. 2024;15(1):18. 10.1186/s13229-024-00600-w. 38698474 10.1186/s13229-024-00600-w PMC11064411

[19] Corsello C, Hus V, Pickles A, Risi S, Cook EH Jr, Leventhal BL, Lord C. Between a ROC and a hard place: decision making and making decisions about using the SCQ. J Child Psychol Psychiatry. 2007;48(9):932–40. 10.1111/j.1469-7610.2007.01762.x. 17714378 10.1111/j.1469-7610.2007.01762.x

[20] Costa J, Moreira A, Moreira P, Delgado L, Silva D. Effects of weight changes in the autonomic nervous system: A systematic review and meta-analysis. Clin Nutr. 2019;38(1):110–26. 10.1016/j.clnu.2018.01.006. 29395374 10.1016/j.clnu.2018.01.006

[21] Coupal KE, Heeney ND, Hockin BCD, Ronsley R, Armstrong K, Sanatani S, Claydon VE. Pubertal Hormonal Changes and the Autonomic Nervous System: Potential Role in Pediatric Orthostatic Intolerance. Front Neurosci. 2019;13:1197. 10.3389/fnins.2019.01197. 31798399 10.3389/fnins.2019.01197 PMC6861527

[22] Croen LA, Zerbo O, Qian Y, Massolo ML, Rich S, Sidney S, Kripke C. The health status of adults on the autism spectrum. Autism. 2015;19(7):814–23. 10.1177/1362361315577517. 25911091 10.1177/1362361315577517

[23] Dhanasekara CS, Ancona D, Cortes L, Hu A, Rimu AH, Robohm-Leavitt C, Payne D, Wakefield SM, Mastergeorge AM, Kahathuduwa CN. Association Between Autism Spectrum Disorders and Cardiometabolic Diseases: A Systematic Review and Meta-analysis. JAMA Pediatr. 2023;177(3):248–57. 10.1001/jamapediatrics.2022.5629. 36716018 10.1001/jamapediatrics.2022.5629 PMC9887535

[24] Dollar JM, Calkins SD, Berry NT, Perry NB, Keane SP, Shanahan L, Wideman L. Developmental patterns of respiratory sinus arrhythmia from toddlerhood to adolescence. Dev Psychol. 2020;56(4):783–94. 10.1037/dev0000894. 31999180 10.1037/dev0000894 PMC8188730

[25] Edmiston EK, Jones RM, Corbett BA. Physiological Response to Social Evaluative Threat in Adolescents with Autism Spectrum Disorder. J Autism Dev Disord. 2016;46(9):2992–3005. 10.1007/s10803-016-2842-1. 27318810 10.1007/s10803-016-2842-1 PMC5140009

[26] Edmiston EK, Muscatello RA, Corbett BA. Altered Pre-Ejection Period Response to Social Evaluative Threat in Adolescents with Autism Spectrum Disorder. Research in Autism Spectrum Disorders. 2017;36:57–65. 10.1016/j.rasd.2017.01.008. 29177005 10.1016/j.rasd.2017.01.008 PMC5699479

[27] El-Sheikh M. Stability of respiratory sinus arrhythmia in children and young adolescents: a longitudinal examination. Dev Psychobiol. 2005;46(1):66–74. 10.1002/dev.20036. 15690389 10.1002/dev.20036

[28] Ellenbroek BA, Sengul HK. Autism spectrum disorders: Autonomic alterations with a special focus on the heart. Heart and Mind. 2017;1(2):78. 10.4103/hm.hm_5_17.

[29] Esteban-Figuerola P, Morales-Hidalgo P, Arija-Val V, Canals-Sans J. Are there anthropometric and body composition differences between children with autism spectrum disorder and children with typical development? Analysis by age and spectrum severity in a school population. Autism. 2021;25(5):1307–20. 10.1177/1362361320987724. 33487005 10.1177/1362361320987724

[30] Eyre EL, Duncan MJ, Birch SL, Fisher JP. The influence of age and weight status on cardiac autonomic control in healthy children: A review. Auton Neurosci. 2014;186:8–21. 10.1016/j.autneu.2014.09.019. 25458714 10.1016/j.autneu.2014.09.019

[31] Fiol-Veny A, De La Torre-Luque A, Balle M, Bornas X. Altered Heart Rate Regulation in Adolescent Girls and the Vulnerability for Internalizing Disorders. Front Physiol. 2018;9:852. 10.3389/fphys.2018.00852. 30038579 10.3389/fphys.2018.00852 PMC6046384

[32] Friedman BH. An autonomic flexibility-neurovisceral integration model of anxiety and cardiac vagal tone. Biol Psychol. 2007;74(2):185–99. 10.1016/j.biopsycho.2005.08.009. 17069959 10.1016/j.biopsycho.2005.08.009

[33] Giuliano RJ, Karns CM, Bell TA, Petersen S, Skowron EA, Neville HJ, Pakulak E. Parasympathetic and sympathetic activity are associated with individual differences in neural indices of selective attention in adults. Psychophysiology. 2018;55(8): e13079. 10.1111/psyp.13079. 29624675 10.1111/psyp.13079 PMC12634002

[34] Greenlee JL, Mosley AS, Shui AM, Veenstra-VanderWeele J, Gotham KO. Medical and Behavioral Correlates of Depression History in Children and Adolescents With Autism Spectrum Disorder. Pediatrics. 2016;137(Suppl 2):S105-114. 10.1542/peds.2015-2851I. 26908466 10.1542/peds.2015-2851I PMC4915738

[35] Guy L, Souders M, Bradstreet L, DeLussey C, Herrington JD. Brief report: emotion regulation and respiratory sinus arrhythmia in autism spectrum disorder. J Autism Dev Disord. 2014;44(10):2614–20. 10.1007/s10803-014-2124-8. 24752681 10.1007/s10803-014-2124-8

[36] Guzzetti S, Magatelli R, Borroni E, Mezzetti S. Heart rate variability in chronic heart failure. Auton Neurosci. 2001;90(1–2):102–5. 10.1016/S1566-0702(01)00274-0. 11485275 10.1016/S1566-0702(01)00274-0

[37] Harder R, Malow BA, Goodpaster RL, Iqbal F, Halbower A, Goldman SE, Fawkes DB, Wang L, Shi Y, Baudenbacher F, Diedrich A. Heart rate variability during sleep in children with autism spectrum disorder. Clinical autonomic research: official journal of the Clinical Autonomic Research Society. 2016;26(6):423–32. 10.1007/s10286-016-0375-5. 27491489 10.1007/s10286-016-0375-5 PMC5106315

[38] Harteveld, L. M., Nederend, I., Ten Harkel, A. D. J., Schutte, N. M., de Rooij, S. R., Vrijkotte, T. G. M., Oldenhof, H., Popma, A., Jansen, L. M. C., Suurland, J., Swaab, H., de Geus, E. J. C., & FemNATCD Consortium. Maturation of the Cardiac Autonomic Nervous System Activity in Children and Adolescents. J Am Heart Assoc. 2021;10(4): e017405. 10.1161/JAHA.120.017405. 33525889 10.1161/JAHA.120.017405 PMC7955328

[39] Hinnant JB, Elmore-Staton L, El-Sheikh M. Developmental trajectories of respiratory sinus arrhythmia and preejection period in middle childhood. Dev Psychobiol. 2011;53(1):59–68. 10.1002/dev.20487. 20882584 10.1002/dev.20487 PMC4004606

[40] Hirvikoski T, Mittendorfer-Rutz E, Boman M, Larsson H, Lichtenstein P, Bolte S. Premature mortality in autism spectrum disorder. Br J Psychiatry. 2016;208(3):232–8. 10.1192/bjp.bp.114.160192. 26541693 10.1192/bjp.bp.114.160192

[41] IBM. (2021). IBM SPSS Statistics for Mac. In (Version Version 28.0) IBM Corp.

[42] Idrees I, Bellato A, Cortese S, Groom MJ. The effects of stimulant and non-stimulant medications on the autonomic nervous system (ANS) functioning in people with ADHD: A systematic review and meta-analysis. Neurosci Biobehav Rev. 2023;144: 104968. 10.1016/j.neubiorev.2022.104968. 36427764 10.1016/j.neubiorev.2022.104968

[43] Jakobsen, J., Hauksson, P., & Vestergaard, P. (1984). Heart rate variation in patients treated with antidepressants. An index of anticholinergic effects? *Psychopharmacology*, *84*(4), 544–548. 10.1007/BF00431464 10.1007/BF00431464 6441956

[44] Jobski K, Hofer J, Hoffmann F, Bachmann C. Use of psychotropic drugs in patients with autism spectrum disorders: a systematic review. Acta Psychiatr Scand. 2017;135(1):8–28. 10.1111/acps.12644. 27624381 10.1111/acps.12644

[45] Kahathuduwa CN, West BD, Blume J, Dharavath N, Moustaid-Moussa N, Mastergeorge A. The risk of overweight and obesity in children with autism spectrum disorders: A systematic review and meta-analysis. Obes Rev. 2019;20(12):1667–79. 10.1111/obr.12933. 31595678 10.1111/obr.12933

[46] Kang K, Seidlitz J, Bethlehem RAI, Xiong J, Jones MT, Mehta K, Keller AS, Tao R, Randolph A, Larsen B, Tervo-Clemmens B, Feczko E, Dominguez OM, Nelson SM, Chart LB, C., Schildcrout, J., Fair, D. A., Satterthwaite, T. D., Alexander-Bloch, A., & Vandekar, S. Study design features increase replicability in brain-wide association studies. Nature. 2024;636(8043):719–27. 10.1038/s41586-024-08260-9. 39604734 10.1038/s41586-024-08260-9 PMC11655360

[47] Kemp AH, Quintana DS. The relationship between mental and physical health: insights from the study of heart rate variability. Int J Psychophysiol. 2013;89(3):288–96. 10.1016/j.ijpsycho.2013.06.018. 23797149 10.1016/j.ijpsycho.2013.06.018

[48] Kemp AH, Quintana DS, Gray MA, Felmingham KL, Brown K, Gatt JM. Impact of depression and antidepressant treatment on heart rate variability: A review and meta-analysis. Biol Psychiat. 2010;67(11):1067–74. 10.1016/j.biopsych.2009.12.012. 20138254 10.1016/j.biopsych.2009.12.012

[49] Kleiger RE, Miller JP, Bigger JT Jr, Moss AJ. Decreased heart rate variability and its association with increased mortality after acute myocardial infarction. Am J Cardiol. 1987;59(4):256–62. 10.1016/0002-9149(87)90795-8. 3812275 10.1016/0002-9149(87)90795-8

[50] Koenig J, Jarczok MN, Warth M, Ellis RJ, Bach C, Hillecke TK, Thayer JF. Body mass index is related to autonomic nervous system activity as measured by heart rate variability–a replication using short term measurements. J Nutr Health Aging. 2014;18(3):300–2. 10.1007/s12603-014-0022-6.24626758 10.1007/s12603-014-0022-6

[51] Koenig J, Kemp AH, Beauchaine TP, Thayer JF, Kaess M. Depression and resting state heart rate variability in children and adolescents - A systematic review and meta-analysis. Clin Psychol Rev. 2016;46:136–50. 10.1016/j.cpr.2016.04.013. 27185312 10.1016/j.cpr.2016.04.013

[52] Koenig J, Rash JA, Campbell TS, Thayer JF, Kaess M. A Meta-Analysis on Sex Differences in Resting-State Vagal Activity in Children and Adolescents. Front Physiol. 2017;8:582. 10.3389/fphys.2017.00582. 28883794 10.3389/fphys.2017.00582 PMC5573740

[53] Kushki A, Brian J, Dupuis A, Anagnostou E. Functional autonomic nervous system profile in children with autism spectrum disorder. Molecular Autism. 2014;5(1):39–10. 10.1186/2040-2392-5-39. 25031832 10.1186/2040-2392-5-39 PMC4099494

[54] Levine TP, Sheinkopf SJ, Pescosolido M, Rodino A, Elia G, Lester B. Physiologic Arousal to Social Stress in Children with Autism Spectrum Disorders: A Pilot Study. Research in Autism Spectrum Disorders. 2012;6(1):177–83. 10.1016/j.rasd.2011.04.003. 22081773 10.1016/j.rasd.2011.04.003 PMC3212393

[55] Liao, D., Cai, J., Rosamond, W. D., Barnes, R. W., Hutchinson, R. G., Whitsel, E. A., Rautaharju, P., & Heiss, G. (1997). Cardiac autonomic function and incident coronary heart disease: a population-based case-cohort study. The ARIC Study. Atherosclerosis Risk in Communities Study. American Journal of Epidemiology, 145(8), 696–706. 10.1093/aje/145.8.696 10.1093/aje/145.8.696 9125996

[56] Lord, C., Rutter, M., DiLavore, P. C., Risi, S., Gotham, K., & Bishop, S. L. (2012). Autism Diagnostic Observation Schedule, 2nd Edition (ADOS-2). Western Psychological Services.

[57] Lozano DL, Norman G, Knox D, Wood BL, Miller BD, Emery CF, Berntson GG. Where to B in dZ/dt. Psychophysiology. 2007;44(1):113–9. 10.1111/j.1469-8986.2006.00468.x. 17241147 10.1111/j.1469-8986.2006.00468.x

[58] Magnon V, Vallet GT, Benson A, Mermillod M, Chausse P, Lacroix A, Bouillon-Minois JB, Dutheil F. Does heart rate variability predict better executive functioning? A systematic review and meta-analysis. Cortex. 2022;155:218–36. 10.1016/j.cortex.2022.07.008. 36030561 10.1016/j.cortex.2022.07.008

[59] Marshall WA, Tanner JM. Variations in pattern of pubertal changes in girls. Arch Dis Child. 1969;44(235):291–303. 10.1136/adc.44.235.291. 5785179 10.1136/adc.44.235.291 PMC2020314

[60] Marshall WA, Tanner JM. Variations in the pattern of pubertal changes in boys. Arch Dis Child. 1970;45(239):13–23. 10.1136/adc.45.239.13. 5440182 10.1136/adc.45.239.13 PMC2020414

[61] Masi CM, Hawkley LC, Rickett EM, Cacioppo JT. Respiratory sinus arrhythmia and diseases of aging: obesity, diabetes mellitus, and hypertension. Biol Psychol. 2007;74(2):212–23. 10.1016/j.biopsycho.2006.07.006. 17034928 10.1016/j.biopsycho.2006.07.006 PMC1804292

[62] Mathewson KJ, Drmic IE, Jetha MK, Bryson SE, Goldberg JO, Hall GB, Santesso DL, Segalowitz SJ, Schmidt LA. Behavioral and cardiac responses to emotional stroop in adults with autism spectrum disorders: influence of medication. Autism Res. 2011;4(2):98–108. 10.1002/aur.176. 21360828 10.1002/aur.176

[63] Matthews KA, Salomon K, Kenyon K, Allen MT. Stability of children’s and adolescents’ hemodynamic responses to psychological challenge: a three-year longitudinal study of a multiethnic cohort of boys and girls. Psychophysiology. 2002;39(6):826–34. 10.1111/1469-8986.3960826. 12462510 10.1111/1469-8986.3960826

[64] McCoy SM, Morgan K. Obesity, physical activity, and sedentary behaviors in adolescents with autism spectrum disorder compared with typically developing peers. Autism. 2020;24(2):387–99. 10.1177/1362361319861579. 31364386 10.1177/1362361319861579

[65] McCraty R, Shaffer F. Heart Rate Variability: New Perspectives on Physiological Mechanisms, Assessment of Self-regulatory Capacity, and Health risk. Global Advances in Integrative Medicine and Health. 2015;4(1):46–61. 10.7453/gahmj.2014.073. 10.7453/gahmj.2014.073 PMC4311559 25694852

[66] Minshew NJ, Turner CA, Goldstein G. The application of short forms of the Wechsler Intelligence scales in adults and children with high functioning autism. J Autism Dev Disord. 2005;35(1):45–52. 10.1007/s10803-004-1030-x. 15796121 10.1007/s10803-004-1030-x

[67] Molfino A, Fiorentini A, Tubani L, Martuscelli M, Rossi Fanelli F, Laviano A. Body mass index is related to autonomic nervous system activity as measured by heart rate variability. Eur J Clin Nutr. 2009;63(10):1263–5. 10.1038/ejcn.2009.35. 19471292 10.1038/ejcn.2009.35

[68] Morgan, E. (2016). *KB0034: How to Use R Peak Detection Settings*. MindWare Technologies, LTD. https://support.mindwaretech.com/knowledge-base/kb0034/?hilite=algorith

[69] Muscatello RA, Andujar J, Taylor JL, Corbett BA. Exploring Key Physiological System Profiles at Rest and the Association with Depressive Symptoms in Autism Spectrum Disorder. J Autism Dev Disord. 2020;51:15–9. 10.1007/s10803-020-04516-1. 10.1007/s10803-020-04516-1 PMC7606213 32350791

[70] Muscatello RA, Kim A, Vandekar S, Corbett BA. Diagnostic and Physical Effects in Parasympathetic Response to Social Evaluation in Youth With and Without Autism Spectrum Disorder. J Autism Dev Disord. 2021. 10.1007/s10803-021-05224-0. 34342805 10.1007/s10803-021-05224-0 PMC8810894

[71] Muscatello RA, Pachol A, Romines A, Smith I, Corbett BA. Development and Parasympathetic Regulation in Male and Female Adolescents with Autism Spectrum Disorder: A Two-Timepoint Longitudinal Study. J Autism Dev Disord. 2022;54:3427–42. 10.1007/s10803-022-05664-2. 10.1007/s10803-022-05664-2 PMC9949914 35829945

[72] Muscatello RA, Vandekar SN, Corbett BA. Evidence for decreased parasympathetic response to a novel peer interaction in older children with autism spectrum disorder: a case-control study. J Neurodev Disord. 2021;13(1):6. 10.1186/s11689-020-09354-x. 33422008 10.1186/s11689-020-09354-x PMC7797088

[73] Must A, Eliasziw M, Phillips SM, Curtin C, Kral TV, Segal M, Sherwood NE, Sikich L, Stanish HI, Bandini LG. The Effect of Age on the Prevalence of Obesity among US Youth with Autism Spectrum Disorder. Child Obes. 2017;13(1):25–35. 10.1089/chi.2016.0079. 27704874 10.1089/chi.2016.0079 PMC5278796

[74] Negriff S, Blankson AN, Trickett PK. Pubertal Timing and Tempo: Associations With Childhood Maltreatment. J Res Adolesc. 2015;25(2):201–13. 10.1111/jora.12128. 26146470 10.1111/jora.12128 PMC4489155

[75] Neuhaus E, Bernier RA, Beauchaine TP. Children with Autism Show Altered Autonomic Adaptation to Novel and Familiar Social Partners. Autism Res. 2016;9(5):579–91. 10.1002/aur.1543. 26305051 10.1002/aur.1543

[76] NHLBI. (2022, 03–24–22). Understand Your Risk for Heart Disease. Retrieved 08–29–24 from

[77] Patriquin MA, Hartwig EM, Friedman BH, Porges SW, Scarpa A. Autonomic response in autism spectrum disorder: Relationship to social and cognitive functioning. Biol Psychol. 2019;145:185–97. 10.1016/j.biopsycho.2019.05.004. 31078720 10.1016/j.biopsycho.2019.05.004

[78] Patriquin MA, Lorenzi J, Scarpa A. Relationship between respiratory sinus arrhythmia, heart period, and caregiver-reported language and cognitive delays in children with autism spectrum disorders. Appl Psychophysiol Biofeedback. 2013;38(3):203–7. 10.1007/s10484-013-9225-6. 23820819 10.1007/s10484-013-9225-6

[79] Pfeifer JH, Allen NB. Puberty Initiates Cascading Relationships Between Neurodevelopmental, Social, and Internalizing Processes Across Adolescence. Biol Psychiat. 2021;89(2):99–108. 10.1016/j.biopsych.2020.09.002. 33334434 10.1016/j.biopsych.2020.09.002 PMC8494463

[80] Porges SW. Social engagement and attachment: a phylogenetic perspective. Ann N Y Acad Sci. 2003;1008(1):31–47. 10.1196/annals.1301.004. 14998870 10.1196/annals.1301.004

[81] Quigley, K. M., & Moore, G. A. (2018). Development of cardiac autonomic balance in infancy and early childhood: A possible pathway to mental and physical health outcomes. Developmental Review, 49, 41–61. 10.1016/j.dr.2018.06.004

[82] Rabbia F, Silke B, Conterno A, Grosso T, De Vito B, Rabbone I, Chiandussi L, Veglio F. Assessment of cardiac autonomic modulation during adolescent obesity. Obes Res. 2003;11(4):541–8. 10.1038/oby.2003.76. 12690083 10.1038/oby.2003.76

[83] Rast JE, Tao S, Schott W, Shea LL, Brodkin ES, Kerns CM, Leonard CE, Murray MJ, Lee BK. Psychotropic Medication Use in Children and Youth with Autism Enrolled in Medicaid. J Autism Dev Disord. 2023. 10.1007/s10803-023-06182-5. 38113012 10.1007/s10803-023-06182-5 PMC11228548

[84] Report of the Council on Science and Public Health (2023). Support Removal of BMI as a Standard Measure in Medicine and Recognizing Culturally-Diverse and Varied Presentations of Eating Disorders and Indications for Metabolic and Bariatric Surgery *CSAPH Report 3-A-24 (Reference Committee D).*

[85] Rutter, M., Bailey, A., & Lord, C. (2003). Social Communication Questionnaire. Western Psychological Services.

[86] Sala M, Lazzaretti M, De Vidovich G, Caverzasi E, Barale F, d’Allio G, Brambilla P. Electrophysiological changes of cardiac function during antidepressant treatment. Ther Adv Cardiovasc Dis. 2009;3(1):29–43. 10.1177/1753944708096282. 19124389 10.1177/1753944708096282

[87] Sammels O, Karjalainen L, Dahlgren J, Wentz E. Autism Spectrum Disorder and Obesity in Children: A Systematic Review and Meta-Analysis. Obes Facts. 2022;15(3):305–20. 10.1159/000523943. 35263756 10.1159/000523943 PMC9210004

[88] Schaaf RC, Benevides TW, Leiby BE, Sendecki JA. Autonomic dysregulation during sensory stimulation in children with autism spectrum disorder. J Autism Dev Disord. 2015;45(2):461–72. 10.1007/s10803-013-1924-6. 23996198 10.1007/s10803-013-1924-6

[89] Schmalenberger, K. M., Eisenlohr-Moul, T. A., Jarczok, M. N., Eckstein, M., Schneider, E., Brenner, I. G., Duffy, K., Schweizer, S., Kiesner, J., Thayer, J. F., & Ditzen, B. (2020). Menstrual Cycle Changes in Vagally-Mediated Heart Rate Variability are Associated with Progesterone: Evidence from Two Within-Person Studies. Journal of Clinical Medicine, 9(3). 10.3390/jcm9030617 10.3390/jcm9030617 PMC7141121 32106458

[90] Sherwood A, Allen MT, Fahrenberg J, Kelsey RM, Lovallo WR, van Doornen LJ. Methodological guidelines for impedance cardiography. Psychophysiology. 1990;27(1):1–23. 10.1111/j.1469-8986.1990.tb02171.x. 2187214 10.1111/j.1469-8986.1990.tb02171.x

[91] Shibao, C., & Okamoto, L. (2012). Agents Potentiating Sympathetic Tone. In D. Robertson, I. Biaggioni, G. Burnstock, P. A. Low, & J. F. R. Paton (Eds.), (3rd ed., pp. 627–630). Academic Press.

[92] Simonoff E, Pickles A, Charman T, Chandler S, Loucas T, Baird G. Psychiatric disorders in children with autism spectrum disorders: prevalence, comorbidity, and associated factors in a population-derived sample. J Am Acad Child Adolesc Psychiatry. 2008;47(8):921–9. 10.1097/CHI.0b013e318179964f. 18645422 10.1097/CHI.0b013e318179964f

[93] Smeekens I, Didden R, Verhoeven EWM. Exploring the relationship of autonomic and endocrine activity with social functioning in adults with autism spectrum disorders. J Autism Dev Disord. 2015;45(2):495–505. 10.1007/s10803-013-1947-z. 24062183 10.1007/s10803-013-1947-z

[94] Stein, C. M. (2012). Beta-Adrenergic Receptors. In D. Robertson, I. Biaggioni, G. Burnstock, P. A. Low, & J. F. R. Paton (Eds.), Primer on the Autonomic Nervous System (Third ed., pp. 59–61). Academic Press.

[95] Task Force of the European Society of Cardiology and The North American Society of Pacing and Electrophysiology. Heart rate variability: standards of measurement, physiological interpretation, and clinical use. Eur Heart J. 1996;17:354–81. 10.1161/01.cir.93.5.1043. 8737210

[96] Thapa R, Alvares GA, Zaidi TA, Thomas EE, Hickie IB, Park SH, Guastella AJ. Reduced heart rate variability in adults with autism spectrum disorder. Autism Res. 2019;12(6):922–30. 10.1002/aur.2104. 30972967 10.1002/aur.2104

[97] Thapa R, Pokorski I, Ambarchi Z, Thomas E, Demayo M, Boulton K, Matthews S, Patel S, Sedeli I, Hickie IB, Guastella AJ. Heart Rate Variability in Children With Autism Spectrum Disorder and Associations With Medication and Symptom Severity. Autism Res. 2021;14(1):75–85. 10.1002/aur.2437. 33225622 10.1002/aur.2437

[98] Thayer JF, Lane RD. Claude Bernard and the heart-brain connection: Further elaboration of a model of neurovisceral integration. Neurosci Biobehav Rev. 2009;33(2):81–8. 10.1016/j.neubiorev.2008.08.004. 18771686 10.1016/j.neubiorev.2008.08.004

[99] Thayer JF, Smith M, Rossy LA, Sollers JJ, Friedman BH. Heart period variability and depressive symptoms: Gender differences. Biol Psychiat. 1998;44(4):304–6. 10.1016/s0006-3223(98)00008-0. 9715364 10.1016/s0006-3223(98)00008-0

[100] Thayer JF, Sternberg E. Beyond Heart Rate Variability: Vagal Regulation of Allostatic Systems. Ann N Y Acad Sci. 2006;1088(1):361–72. 10.1196/annals.1366.014. 17192580 10.1196/annals.1366.014

[101] Thayer JF, Yamamoto SS, Brosschot JF. The relationship of autonomic imbalance, heart rate variability and cardiovascular disease risk factors. Int J Cardiol. 2010;141(2):122–31. 10.1016/j.ijcard.2009.09.543. 19910061 10.1016/j.ijcard.2009.09.543

[102] Triggiani AI, Valenzano A, Ciliberti MA, Moscatelli F, Villani S, Monda M, Messina G, Federici A, Babiloni C, Cibelli G. Heart rate variability is reduced in underweight and overweight healthy adult women. Clin Physiol Funct Imaging. 2017;37(2):162–7. 10.1111/cpf.12281. 26211739 10.1111/cpf.12281

[103] Tsuji H, Larson MG, Venditti FJ Jr, Manders ES, Evans JC, Feldman CL, Levy D. Impact of reduced heart rate variability on risk for cardiac events. The Framingham Heart Study Circulation. 1996;94(11):2850–5. 10.1161/01.cir.94.11.2850. 8941112 10.1161/01.cir.94.11.2850

[104] van Steensel FJA, Bögels SM, Perrin S. Anxiety disorders in children and adolescents with autistic spectrum disorders: a meta-analysis. Clin Child Fam Psychol Rev. 2011;14(3):302–17. 10.1007/s10567-011-0097-0. 21735077 10.1007/s10567-011-0097-0 PMC3162631

[105] van Zyl LT, Hasegawa T, Nagata K. Effects of antidepressant treatment on heart rate variability in major depression: a quantitative review. Biopsychosocial Medicine. 2008;2:12. 10.1186/1751-0759-2-12. 18590531 10.1186/1751-0759-2-12 PMC2478652

[106] Vandekar S, Tao R, Blume J. A Robust Effect Size Index Psychometrika. 2020;85(1):232–46. 10.1007/s11336-020-09698-2. 32232646 10.1007/s11336-020-09698-2 PMC7186256

[107] Vaughan Van Hecke A, Lebow J, Bal E, Lamb D, Harden E, Kramer A, Denver J, Bazhenova O, Porges SW. Electroencephalogram and heart rate regulation to familiar and unfamiliar people in children with autism spectrum disorders. Child Dev. 2009;80(4):1118–33. 10.1111/j.1467-8624.2009.01320.x. 19630897 10.1111/j.1467-8624.2009.01320.x

[108] Wagner NJ, Holochwost SJ, Lynch SF, Mills-Koonce R, Propper C. Characterizing change in vagal tone during the first three years of life: A systematic review and empirical examination across two longitudinal samples. Neurosci Biobehav Rev. 2021;129:282–95. 10.1016/j.neubiorev.2021.07.025. 34324920 10.1016/j.neubiorev.2021.07.025 PMC8429175

[109] Walsh, C. A. (2001). Syncope and sudden death in the adolescent. Adolescent Medicine, 12(1), 105–132. https://www.ncbi.nlm.nih.gov/pubmed/11224026 11224026

[110] Watson LR, Roberts JE, Baranek GT, Mandulak KC, Dalton JC. Behavioral and physiological responses to child-directed speech of children with autism spectrum disorders or typical development. J Autism Dev Disord. 2012;42(8):1616–29. 10.1007/s10803-011-1401-z. 22071788 10.1007/s10803-011-1401-z PMC3402684

[111] Wechsler, D. (2011). *Wechsler Abbreviated Scale of Intelligence, 2nd Edition (WASI-II)* (2nd ed.). Pearson.

[112] WHO. (2021, 06–11–2021). *Cardiovascular diseases (CVDs)*. Retrieved 08–29–2024 from https://www.who.int/news-room/fact-sheets/detail/cardiovascular-diseases-(cvds)

